# CASTER: Cross-Sectional Asthma STEroid Response Measurement

**DOI:** 10.3390/jpm10030095

**Published:** 2020-08-20

**Authors:** Alvin T. Kho, Joanne Sordillo, Ann Chen Wu, Michael H. Cho, Sunita Sharma, Anshul Tiwari, Jessica Lasky-Su, Scott T. Weiss, Kelan G. Tantisira, Michael J. McGeachie

**Affiliations:** 1 Computational Health Informatics Program, Boston Children’s Hospital, Boston, MA 02215, USA; alvin_kho@hms.harvard.edu; 2 PRecisiOn Medicine Translational Research (PROMoTeR) Center, Department of Population Medicine, Harvard Pilgrim Health Care Institute and Harvard Medical School, Boston, MA 02215, USA; rejoa@channing.harvard.edu (J.S.); ann.wu@childrens.harvard.edu (A.C.W.); 3 Channing Division of Network Medicine, Department of Medicine, Brigham and Women’s Hospital and Harvard Medical School, Boston, MA 02215, USA; remhc@channing.harvard.edu (M.H.C.); reati@channing.harvard.edu (A.T.); jessica.a.su@gmail.com (J.L.-S.); restw@channing.harvard.edu (S.T.W.); rekgt@channing.harvard.edu (K.G.T.); 4 Department of Medicine—Pulmonary Sciences and Critical Care Medicine, University of Colorado Denver, Aurora, CO 80010, USA; sunita.sharma@ucdenver.edu

**Keywords:** asthma, steroid response, inhaled corticosteroids, pharmacogenetics

## Abstract

Asthma patient response to inhaled corticosteroids (ICS) is variable and difficult to quantify. We aimed to define a measure of steroid response suitable for pharmacogenetic research in longitudinal and cross-sectional cohorts. Using longitudinal data from the Childhood Asthma Management Program (CAMP) asthma cohort, we defined the Cross-sectional Asthma STEroid Response (CASTER) measure in cross-sectional data. We then applied this to cross-sectional slices of four independent asthma cohorts: The Improving Asthma Control Trial (IMPACT), the Salmeterol or Corticosteroids Study (SOCS), the Pediatric Asthma Controller Trial (PACT), and the Genetics of Asthma in Costa Rica Study (GACRS). CASTER achieved high accuracy on the childhood asthma cohorts: GACRS, PACT, and also on cross-sectional data from CAMP (AUCs 82%, 71%, 63%, respectively). This demonstrates that select cross-sectional clinical information is sufficient to identify good and poor responders to ICS treatment in childhood asthma. Thus, CASTER represents a major improvement in the usability and applicability of steroid response measures in asthma research.

## 1. Introduction

Inhaled corticosteroids (ICS) are the most commonly prescribed controller medications for asthma. Although ICS are among the most effective medications for preventing future asthma exacerbations, an estimated 25–30% of patients do not respond to ICS therapy [1,2]. Steroid-resistant asthma is generally more severe, and, by virtue of being resistant to ICS, harder to treat, and thus results in a greater proportion of the costs incurred nationally by asthma morbidity [3]. Steroid-resistant asthma is etiologically different from allergic asthma in that it is usually not a result of type-2 cytokine inflammation. This can occur through a number of mechanisms, including malfunction of the glucocorticoid receptor [4] and viral or bacterial respiratory infections [5]. The mechanisms of steroid resistance have been recently reviewed by Wadwha et al. [5].

Clinical asthma management would benefit from a method to identify patients who are poor responders to ICS early on, rather than the current standard of care which includes trial-and-error; only after ICS treatment appears ineffective would other medications be added or substituted for the ICS. Nevertheless, a precise quantitative definition of steroid response has been difficult to obtain; intuitively, someone on ICS who continues to have asthma exacerbations and morbidity would be considered a non-responder, but recent work showed that a more detailed approach is more effective. Clemmer et al. [6] defined a measure of ICS response by combining: (1) frequencies of courses of oral steroid bursts, (2) asthma-related hospitalizations or emergency department (ED) visits, (3) bronchodilator response, (4) methacholine challenge, (5) forced expiratory volume in one second (FEV1), and (6) nocturnal asthma-related awakenings. They showed that this approach provided a more accurate indicator of steroid response than any of these data alone.

The method of Clemmer et al. [6] measures the Steroid Responsiveness Endophenotype (SRE), and was effective in distinguishing steroid response in several independent cohorts. Nevertheless, that method has limitations. Most importantly, it is an inherently longitudinal measure, requiring observation over a period of ICS use. A measure of the SRE for cross-sectional data, or at least data obtained from a single visit with a provider, would be more widely used in research. With a cross-sectional measure of SRE, it could be applied to many cross-sectional asthma cohorts and used by researchers to identify which patients benefit most from ICS treatment. After ICS responders and non-responders are identified, investigations can be conducted using DNA variants, RNA transcripts, or clinical or demographic factors most associated with good or poor ICS response. We stress here that our goal is to define steroid response, not to predict who will respond well to steroids. Before work can be conducted to predict who responds to ICS treatment, we must define, in as satisfactory a way possible, what it means to “respond to ICS treatment”.

We propose here the Cross-sectional Asthma STEroid Response (CASTER) measure of ICS response, which has a number of benefits. First, it is defined over cross-sectional data, which is more readily available. Additionally, CASTER has similar accuracy to the more complicated Clemmer et al. [6] SRE measure. Finally, since ICS response can change in an individual with time, a cross-sectional approach can have benefits compared with a longitudinal measure considered over a long period. The code to compute CASTER is available by anonymous download at http://www.cgbayesnets.com/caster.html. This work was approved by the Partners Healthcare institutional review board (protocol #2017P001799) and informed consent was obtained for all participants.

## 2. Materials and Methods

### 2.1. Cohorts

The Childhood Asthma Management Program (CAMP) was a multi-center, randomized, double-blinded, clinical trial of inhaled corticosteroids. CAMP comprised 1041 children with mild-to-moderate persistent asthma, from ages 5–12, who were followed for four years. The design [7] and outcomes [8] of this study have been previously reported.

The Genetics of Asthma in Costa Rica Study (GACRS) was a cross-sectional study that recruited from February 2001 to August 2008. Questionnaires were sent to the parents of 16,912 children (ages 6–14 years) enrolled in 140 Costa Rican schools; 9180 (54.3%) questionnaires were returned. Children were eligible for the study if they had asthma, defined as a physician diagnosis and two or more respiratory symptoms or asthma attacks in the prior year, and a high probability of having six or more great-grandparents born in the Central Valley of Costa Rica, which was determined by a genealogist on the basis of the paternal and maternal last names of each of the child’s parents. Of the 9180 children screened, 3113 (33.9%) had asthma. Of these, a total of 1165 asthmatic children were enrolled. Protocols and assessments in GACRS have been previously published [9], but included FEV1, bronchodilator response (BDR), methacholine challenge, and questionnaires about previous medication use and exacerbations.

The Improving Asthma Control Trial (IMPACT) was a randomized trial that enrolled 225 adults [10]. Participants were randomized into three arms: an ICS (budesonide); a leukotriene antagonist (LTRA, zafirlukast); or placebo. Additionally, patients had access to open-label budesonide for exacerbation management. For the current investigation, we used data from the subjects completing the trial who had completed spirometry measurements (n = 149). During the clinical trial, IMPACT also collected data on exacerbations, hospitalizations, BDR, and methacholine challenge.

The Salmeterol or Corticosteroids Study (SOCS) [11] enrolled adult subjects with moderate persistent asthma who received ICS (triamcinolone) for a six-week run-in period prior to randomization. We used data from this six-week period, which included exacerbations, hospitalizations, a methacholine challenge, and FEV1. There was no bronchodilator assessment performed.

The Pediatric Asthma Controller Trial (PACT) was a 48-month randomized clinical trial of asthma controller medications, with three arms: ICS alone; ICS in combination with inhaled Long-Acting Beta Agonist (LABA); or a leukotriene receptor agonist (LTRA) alone [12]. Children aged 6–14 years with mild-moderate persistent asthma were recruited and randomized. Assessments included FEV1, change in BDR, methacholine challenge and catalogs of exacerbations and hospitalizations.

### 2.2. Clinical Measurements

Our analysis included five clinical measurements from each of the trials: (1) FEV1 percent predicted as a percentage of the predicted FEV1 for a person of the same age, sex, height, and race according to established models [13]. (2) BDR computed as a percentage change from pre-bronchodilator FEV1: (post-FEV1 − pre-FEV1)/pre-FEV1. (3) Airway hyperresponsiveness assessed by a methacholine challenge and measured as the Percentage Concentration of methacholine necessary to effect a 20% reduction in FEV1 (PC20). (4) Supplemental systemic oral steroid courses (bursts) required for asthma exacerbation management. (5) Asthma-related hospitalizations and/or ED visits.

We focused on obtaining a measure of SRE that worked with cross-sectional data: data obtainable from a single visit or interaction with a health care provider. In the CAMP cohort, we took cross-sectional data from the end of year-one of the clinical trial. In the other clinical trial cohorts, we took cross sectional data from the end of the clinical trial, including previous counts of oral steroid bursts and hospitalizations. The GACRS cohort is a cross-sectional study by design.

For purposes of comparison, we also considered an SRE measure based on longitudinal data from these clinical trial cohorts, by using the changes from baseline to the trial end in BDR, FEV1, and PC20, and by using the total number of bursts and hospitalizations throughout each trial.

### 2.3. Formulation of Poor and Good Responder Representative Profiles

CAMP was our discovery cohort for defining the CASTER measure of poor and good ICS response. We studied the 311 subjects randomized to ICS. We split the subjects into two groups based upon their cumulative supplemental oral steroid (prednisone) courses (3–5 day courses of systemic steroids, called “bursts”) in year 1: the poor responder group (N = 74) with two or more bursts, and the good responder group (N = 237) with one or zero bursts. To characterize these groups more broadly across clinical and spiro-metric measures, we examined the other four clinical characteristics (above). We averaged FEV1 over the first year of treatment subsequent to baseline measurement, comprising six measurements in total. This average was compared to the baseline FEV1 at randomization and converted to a percentage change of the baseline amount. We then compared the changes in two assessments of airway hyperreactivity, the first at a clinical visit just before randomization, and then at a visit eight months after randomization. We investigated the change in natural log PC20 (LnPC20) from these two assessments. We averaged BDR over the first year of treatment subsequent to baseline measurement, as with FEV1. Finally, asthma-related ED visits and/or hospitalizations were summed. Missing values for spiro-metric variables were imputed using their respective population median.

We then computed quartiles of each of these five measures (Table 1). These quartiles were then averaged to arrive at a single “centroid” of the good and poor distributions, respectively (Table 2).

### 2.4. Partial Least-Squares Regression

Using the first year of longitudinal CAMP data on the five clinical variables (oral steroid bursts, asthma-related ED visits and/or hospitalizations, FEV1, LnPC20, BDR, as above), we performed a partial least-squares (PLS) regression to identify meaningful linear combinations of these while maximizing the covariance with ICS use. CAMP data were centered before PLS-regression. We used PLS regression as implemented in MATLAB R2018a (Mathworks, Natick, MA, USA), using the SIMPLS algorithm [14] with default parameters. The first three PLS components are shown in Table 2. As in Principal Component Analysis (PCA), these PLS components can be used to project data from the CAMP cohort or replication cohorts into PLS space, by matrix multiplication.

To make a comparison between dimensionality reduction projection methods, we also computed the same measures using PCA and compared to our PLS version.

### 2.5. Classification by PLS Distance to Response Categories

We then projected the averaged quartiles of good and poor responders, respectively, into PLS-components, to obtain projections of good and poor centroids. These were then used to classify subjects as either likely to be “poor responders” or “good responders” based on their Euclidean distance to each of these centroids in PLS-space.

Classification of each subject as a “good responder” or “poor responder” proceeded by cohort, with each cohort being projected into the space defined by the first three PLS-components, and then distance to good and poor centroids used as the classification for ICS response. To use cross-sectional rather than longitudinal data, we centered LnPC20 and BDR within each cohort and normalized FEV1.

### 2.6. Evaluating the CASTER Measure of SRE

We defined the CASTER measure of SRE based on differences between good and poor ICS responder groups in CAMP, but to evaluate it we needed an orthogonal metric. It would be natural to assume that indicators of a good response to ICS include a decrease in cumulative bursts and/or an increase in FEV1 over time. However, since cumulative bursts and FEV1 are constituent clinical features in the formulation of CASTER, it would be tautological to evaluate CASTER against either feature. Therefore, as with previous work [6], we investigated the ability of CASTER to discriminate between the ICS-treated vs. non-ICS-treated groups in each cohort. In this way, we use ICS treatment as a proxy for SRE since participants are randomized to ICS treatment and an estimated 70–75% of asthmatics respond to ICS [1]. This gives us a reasonable proxy for SRE in the clinical trials without defining an independent measure of ICS response, since most of the subjects randomized to ICS will respond well to ICS, and the subjects randomized to placebo or less-effective treatments will not be responding to ICS.

As in previous work, to increase sample size we included non-ICS drugs in the non-ICS group and ICS combination therapy in the ICS group [6]. LABAs were grouped with placebos in the SOCS cohort. Salmeterol/ICS combination therapy was grouped with ICS-only therapy in PACT. There was no placebo treatment available in PACT, therefore we considered the non-ICS group to be those participants given a LTRA (montelukast).

Since the GACRS cohort was the only cohort that was not a clinical trial, there was confounding by indication: ICS use was strongly correlated with an increase in exacerbations. We therefore restricted our investigation only to the GACRS subjects on ICS and used asthma-related hospitalizations as the proxy for SRE (one or more hospitalization indicating poor SRE), while excluding those hospitalizations from the CASTER computation in GACRS.

## 3. Results

As our discovery population we used the children of CAMP. Four replication populations were IMPACT, PACT, GACRS, and SOCS (Table 3). Our replication populations included both children (PACT and GACRS) and adults (SOCS and IMPACT), clinical trials (PACT, SOCS, IMPACT) and cross-sectional ascertainments (GACRS). Subjects in individual clinical trials were limited to a single ICS drug, although across populations a variety of different ICS were used. Across all populations, subjects had relatively normal lung function (mean FEV1 percentage predicted from 90.4% to 101.9%), except SOCS at 85% FEV1. BDR was relatively consistent across the populations (mean BDR 5.5% to 9.8%), as was airway responsiveness (mean LnPC20 0.47 to 0.78). Bursts was more variable with CAMP (1.4 +/− 1.8) and GACRS (1.9 +/− 0.66) having more than PACT (0.88 +/− 1.0), and IMPACT (.17 +/− 0.43) and SOCS (0.17 +/− 0.42) having much fewer.

We initially tried predicting the GACRS cross-sectional cohort as we did the clinical studies. But since GACRS was a population sample (with inclusion criteria of a history of asthma), ICS use was confounded with oral steroid bursts. It appeared in this cohort that ICS use led to greater bursts since the subjects using ICS were suffering from more serious asthma. We therefore limited attention only to the GACRS participants on ICS and had to choose a different proxy for the SRE in GACRS. To be closest to the purpose of the CASTER measure when measuring control on ICS, we chose hospitalizations as the proxy of SRE.

Results of predicting SRE proxy in each cohort are shown in Table 4. Cross sectional data was our major interest. Using cross-sectional data, CASTER was able to differentiate SRE proxy in PACT (0.71 AUC, 95% CI (0.63–0.80)) and in GACRS (0.82 AUC, 95% CI (0.76–0.88)). Performance in IMPACT and SOCS was not significantly different from random guessing. While the model was trained on CAMP longitudinal data, the performance on CAMP cross-sectional data was good (0.63 AUC, 95% CI (0.59–0.66)). For comparison, we also tested the longitudinal cohorts using longitudinal data, and found slight improvements in CAMP and PACT (Table 2).

To make a more straightforward comparison with previous work, we also computed CASTER measures using principal components analysis rather than partial-least squares regression to transform the clinical data and project the response-category quartile centroids. This resulted in uniformly poorer performance on cross-sectional data in every cohort, with particular decline in GACRS (0.51 AUC, not significantly different from random).

## 4. Discussion

Our study has three key findings. First, using cross-sectional data, CASTER can distinguish good and poor responders to ICS. Secondly, CASTER has high accuracy in pediatric studies, similar to the accuracy of previous SRE measures for longitudinal studies. Third, CASTER is a measure of steroid response that is suitable for pharmacogenetic research in cross-sectional studies.

We developed CASTER using five cross-sectional measures: oral steroid courses; asthma-related hospitalizations and ED visits; pre-bronchodilator FEV1; PC20; and BDR. Following the approach of Clemmer et al. [6], we tested our CASTER as a definition of ICS response on cohorts with subjects on and off ICS. We consider the subjects on ICS to be “responders” and the subjects not on ICS to be “non-responders.” This stance includes some false-positives, in that some of the ICS subjects (estimated 25–30%) are expected to be non-responders; however it includes no false negatives as subjects not given ICS cannot be responding well to a treatment not provided. In this way, our estimates of the accuracy of the CASTER measure of ICS response is a lower-bound, but it is directly comparable with the SRE measure of Clemmer et al. [6].

CASTER has high accuracy that is similar to the Clemmer et al. [6] SRE measure. Because that measure was computed using longitudinal data and included asthma symptoms measured by daily diaries, which is inherently longitudinal, some may expect the Clemmer et al. measure to be more accurate. Nevertheless, accuracy of these authors’ SRE measure was relatively consistent at 0.69 to 0.73 AUC across replication cohorts [6], which is comparable to CASTER performance in the childhood cohorts PACT and GACRS (Table 2). CASTER performance in the adult cohorts, IMPACT and SOCCS, was poorer. This may be due to biological differences between adult and childhood asthma. We speculate that perhaps the measures CASTER learned from the childhood CAMP cohort are somehow more reproducible in other childhood cohorts, even cross-sectional childhood studies.

Longitudinal studies are costly and time-consuming; thus, being able to determine ICS responsiveness using cross-sectional data has significant value for pharmacogenetic studies. Thus, our development of CASTER, which defines ICS response using cross-sectional data, will have great implications for future pharmacogenetic research. Being able to use cross-sectional data to determine ICS response has significant advantages, and CASTER accounts for multiple aspects of ICS response beyond what each component predicts. Furthermore, the populations studied were significantly different as shown in Table 3, increasing the generalizability of CASTER to other populations, with significant differences in all the demographic and clinical features across cohorts. These differences remained statistically significant when limited only to the childhood cohorts, PACT and GACRS, indicating strong generalization among the cohorts with high SRE classification accuracy. CASTER may have been weakened by learning the SRE measure on only one childhood cohort; with access to many more cohorts, a stronger methodology may result in a better metric. It is also possible that measures of steroid response could be defined using fewer, or altogether separate, clinical variables. Our work here was based closely on Clemmer et al., which showed that six features were superior to any one separately, but future work could seek to expand or contract our results.

Despite the strengths of our study in developing a cross-sectional definition of ICS response rather than a longitudinal one, there may be objections that we have not measured a true ICS response. In order to do so, we should measure asthma control before ICS, and during a specific window of ICS treatment, and then compare the change to other patients, taking into consideration initial asthma severity. While this may provide a truer measure of ICS response, it would violate two of our main goals: (1) being cross-sectional; and (2) being generally easier to apply in a wider range of cohorts and situations. By measuring current lung function on ICS (BDR, methacholine challenge, and FEV1), and using self-reported recent exacerbations on ICS, we may have created an absolute measure of asthma severity while on ICS. This measures asthma control and lung function on ICS, and compares it to an objective standard of goodness for these attributes—fewer exacerbations is better, greater lung function is preferred, and these regardless of how severe or mild these measures were prior to ICS treatment for a given patient. This has the effect of identifying patients who are not sufficiently controlled on ICS and are thus the ones that will require a step-up in therapy. Under this interpretation, CASTER is still a particularly useful measurement. We have also shown that it is effective, and statistically similar to previous SRE measures [6]. In addition, the proxy of SRE in GACRS was hospitalizations. However, previous work has shown that using any one clinical phenotype as a measure of SRE was less accurate that using a composite. We therefore do not consider hospitalizations on ICS treatment to be the ultimate measure of ICS response, even in GACRS, since there may be subjects responding through other clinical measures but experiencing a hospitalization, and vice versa.

In summary, select cross-sectional clinical information is sufficient to identify good and poor responders to ICS treatment in childhood asthma. CASTER represents a major improvement in the usability and applicability of steroid response measures and will pave the way for future asthma research in steroid responsiveness.

## Figures and Tables

**Table 1:** Quartiles of the Good and Poor responder groups of the inhaled corticosteroids (ICS) treatment arm of the Childhood Asthma Management Program (CAMP) trial.

	Poor (n = 74)			Good (n = 237)		
	25th Percentile	50th Percentile	75th Percentile	25th Percentile	50th Percentile	75th Percentile
Bursts	3	3	6	0	0	0
ED/Hosps	0	0	1	0	0	0
FEV1	−4.3	1.8	8.65	−0.077	3.38	10.7
LnPC20	0.163	0.55	1.09	0.46	1.03	1.64
BDR	5	6.7	11	4.5	6.1	8.2

Bursts: Number of supplemental oral steroid courses required for treatment. ED/Hosps: Number of asthma-related emergency department visits or hospitalizations. FEV1: Forced Expiratory Volume in 1 second, given as a percent of predicted. LnPC20: natural logarithm of provocative concentration of methacholine. BDR: Bronchodilator response to Albuterol, percentage change in FEV1.

**Table 2:** Loadings of the first three PLS components from CAMP, with projections of the Good and Poor responder centroids into this space.

	PLS Comp 1	PLS Comp 2	PLS Comp 3
BURSTS	0	−0.0163	0.00402
ED/Hosps	0	−0.00321	−0.000574
FEV1	0.000072	−0.000003	−0.000007
LnPC20	0	0.00621	0.0274
BDR	0	−0.000537	−0.00241
poor centroid	0.000147	−0.0625	0.0323
good centroid	0.000339	0.00646	0.0285

Bursts: Number of supplemental oral steroid courses required for treatment. ED/Hosps: Number of asthma-related emergency department visits or hospitalizations. FEV1: Forced Expiratory Volume in 1 second, given as a percent of predicted. LnPC20: natural logarithm of provocative concentration of methacholine. BDR: Bronchodilator response to Albuterol, percentage change in FEV1.

**Table 3:** Population Characteristics.

	CAMP	IMPACT	SOCS	PACT	GACRS	*p*-Value
N	1041	149	75	150	594	
ICS, n (%)	311 (29.9%)	53 (35.6%)	23 (30.7%)	99 (66%)	76 (12.8%) *	2.80 × 10^−38^
Age, years (+/−std)	8.9 (+/−2.1)	33.5 (+/−10.5)	30.7 (+/−10.8)	9.7 (+/−2.0)	9.2 (+/−1.9)	0
Sex, n male (%)	621 (59.7%)	54 (36.2%)	27 (36%)	88 (62.4%)	251 (42.3%)	1.40 × 10^−15^
Oral steroid bursts, mean (+/−std)	1.4 (+/−1.8)	0.17 (+/−0.43)	0.17 (+/−0.42)	0.88 (+/−1.0)	1.9 (+/−0.66)	7.30 × 10^−54^
ED visits/Hospitalizations, mean (+/−std)	0.3 (+/−0.82)	0.09 (+/−0.28)	0.16 (+/−0.37)	0.25 (+/−0.52)	NA	0.007
FEV1, mean (+/−std)	95.2 (+/−13.6)	90.4 (+/−12.5)	85.5 (+/−14.4)	101.9 (+/−10.7)	97.6 (+/−17.7)	4.20 × 10^−19^
LnPC20, mean (+/−std)	0.47 (+/−1.3)	0.58 (+/−1.4)	0.60 (+/−0.57)	0.50 (+/−1.5)	0.78 (+/−0.44)	6.90 × 10^−6^

CAMP: The Childhood Asthma Management Program. GACRS: The Genetics of Asthma in Costa Rica Study. IMPACT: The Improving Asthma Control Trial. SOCS: The Salmeterol or Corticosteroids Study. PACT: The Pediatric Asthma Controller Trial. FEV1: Forced Expiratory Volume in 1 second, given as a percent of predicted. Ln PC20: logarithm of provocative concentration of methacholine. BDR: Bronchodilator response to Albuterol, percentage change in FEV1. * In GACRS all subjects were on ICS, and we report here the number with at least one asthma-related ED visit/hospitalization, which was used as the proxy for ICS response. *p*-values computed with ANOVA.

**Table 4:** Accuracy of Steroid Responsiveness Endophenotype (SRE) measure assessment by Cross-sectional Asthma STEroid Response Management (CASTER) methodology vs. Steroid Responsiveness Endophenotype (SRE) proxy.

	Partial Least-Squares (PLS) Components		Principal Components	
Study	Cross Sectional	Longitudinal	Cross Sectional	Longitudinal
CAMP	0.63 ([0.59–0.66]) *	0.68 ([0.65–0.72])	0.63 ([0.59–0.66])	0.65 ([0.61–0.69])
IMPACT	0.59 ([0.49–0.69])	0.55 ([0.46–0.65])	0.53 ([0.43–0.62])	0.55 ([0.45–0.65])
SOCS	0.60 ([0.45–0.75])	0.51 ([0.36–0.66])	0.59 ([0.44–0.75])	0.43 ([0.28–0.58])
PACT	0.71 ([0.63–0.80]) *	0.74 ([0.65–0.82])	0.65 ([0.56–0.75])	0.69 ([0.59–0.79])
GACRS	0.82 ([0.76–0.88]) *	NA	0.51 ([0.44–0.58])	NA

Measurements shown in Area Under Receiver Operator Characteristic Curve (AUC) with 95% confidence intervals. * indicates performance significantly different from random using Cross-Sectional data with a PLS-based model.
